# Bacteria increase host micronutrient availability: mechanisms revealed by studies in *C. elegans*

**DOI:** 10.1186/s12263-020-00662-4

**Published:** 2020-03-05

**Authors:** Claire Maynard, David Weinkove

**Affiliations:** Department of Biosciences, Durham University, Durham, UK

**Keywords:** Micronutrients, Host-microbe model, Folic acid

## Abstract

Micronutrients cannot be synthesized by humans and are obtained from three different sources: diet, gut microbiota, and oral supplements. The microbiota generates significant quantities of micronutrients, but the contribution of these compounds to total uptake is unclear. The role of bacteria in the synthesis and uptake of micronutrients and supplements is widely unexplored and may have important implications for human health. The efficacy and safety of several micronutrient supplements, including folic acid, have been questioned due to some evidence of adverse effects on health. The use of the simplified animal-microbe model, *Caenorhabditis elegans*, and its bacterial food source, *Escherichia coli,* provides a controllable system to explore the underlying mechanisms by which bacterial metabolism impacts host micronutrient status. These studies have revealed mechanisms by which bacteria may increase the bioavailability of folic acid, B12, and iron. These routes of uptake interact with bacterial metabolism, with the potential to increase bacterial pathogenesis, and thus may be both beneficial and detrimental to host health.

## Background

### Dietary, bacterial, and supplemental micronutrients

An array of essential vitamins and minerals, collectively known as micronutrients, is required to drive fundamental biosynthetic cellular reactions. As humans like all animals are unable to synthesize micronutrients, they are acquired from different sources and absorbed along the gastrointestinal (GI) tract; the small intestine primarily absorbs exogenous micronutrients provided by the diet, whereas the large intestine, where density and diversity of the gut microbiota is highest, absorbs endogenous bacterially-derived micronutrients. Several micronutrients are synthesized de novo by the microbiota, notably B-vitamins and vitamin K [1]. The pool of folate (vitamin B9) in the human colon has been described as in excess of dietary intakes [2, 3], and studies have demonstrated that folates can be absorbed across the human colon and assimilated into host tissues [4–6]. The relative contribution of the microbiota to folate status is unclear.

The relationship between diet, microbiota, and host health is complex. With respect to folate, diet plays both a direct and indirect role on host micronutrient status; for example, a diet rich in fiber has been shown to increase colonic folate levels, presumably by increasing the abundance of folate synthesizing obligate anaerobic bacteria in the colon [7]. Recent metagenomic studies have probed the B-vitamin biosynthetic capacity of the human gut microbiota and are consistent with a model in which micronutrients are exchanged between microbial producers and non-producers in synthesis and salvage pathways [8, 9]. This exchange is predicted to play an important role in the stabilization of the microbial community and the maintenance of host-microbe homeostasis. Given the emerging importance of microbiota composition and stability on human health, combined with the impact of diet on bacterial metabolism, it is surprising that the interaction of the third absorbable source of micronutrients, oral supplements, with bacterial metabolism has not been considered.

Inadequate acquisition of just one micronutrient can cause a systemic deficiency. Micronutrient deficiency is estimated to affect two billion people worldwide. Folate deficiency disproportionately affects women and children, due to an increased demand for folate during developmental processes. Folic acid supplements are administered to prevent or ameliorate the symptoms of disorders associated with folate deficiency, notably congenital birth defects such as neural tube defects (NTDs) [10, 11]. Over 60 countries have adopted folic acid fortification programs with the aim of a blanket increase in folate status across the population [12]. While these programs have been successful in decreasing the incidence of births with neural tube defects [13–15], folic acid fortification remains controversial due to associated adverse health outcomes including zinc deficiency caused by impaired absorption in the intestine [16], neurological damage due to masking of the signs of B12 deficiency [17], and an increased risk of colorectal cancer [18]. Studies in rodents have indicated that over-supplementation of folic acid disrupts embryonic development [19, 20]. The safety of folic acid supplementation is still debated, with independent meta-analyses reaching different conclusions [21–23], and the mechanism of action remains unresolved.

It has been hypothesized that supplementation may have a negative effect on health in susceptible individuals or those with an existing high micronutrient status, but it is not clear whether this can be attributed to folic acid itself or resulting high levels of serum folate. A third possibility is the impact of folic acid on microbial metabolism. It is surprising how little is known about how folic acid and how other micronutrient supplements influence bacterial metabolism in the microbiota [24]. Other micronutrient supplements also have reported negative health consequences. For example, iron supplementation is associated with increased risk of colorectal cancer and other intestinal disorders [25]. Interestingly, studies in rodent models of colitis [26, 27], patients with intestinal bowel disease (IBD) [28], and infants [29] have revealed an increased abundance of inflammatory-associated bacterial species and increased markers of inflammation in the intestine following iron supplementation.

A better understanding of the contribution of the microbiota to micronutrient status and its interaction with dietary and supplemental sources is vital but problematic in humans due to the inter-individual variation and complexity of the microbiota, the difficulty in measuring trace amounts of specific micronutrients, and in distinguishing between micronutrients derived from diet, gut microbiota, and supplements. Establishing relevant animal models is important in order to separate the effects of different micronutrient sources on host health, to understand their interactions, and to examine the safety of supplementation in different backgrounds.

#### *C. elegans* relies on *E. coli* metabolism for its micronutrient supply

Significant advances in our understanding have been made using the simplified host-microbe model of the nematode, *Caenorhabditis elegans*, and its non-pathogenic bacterial food-source, *Escherichia coli,* a bacterium which is found in the human gut microbiota. During development and in young adult worms, the host-microbe relationship is primarily nutritional; *E. coli* cells are ingested and masticated in a specialized organ called the pharynx and passed into the intestinal lumen, where macro- and micronutrients from *E. coli* are absorbed across the epithelium and into the pseudocoelom (body cavity) [30]. With aging comes a breakdown of the function of the pharynx and intestinal immunity, allowing *E. coli* cells to colonize the intestine, shifting the host-microbe relationship [31]. Maintenance of worms on axenic media or with metabolically inactive *E. coli* slows *C. elegans* development and adversely affects reproductive fitness, indicating a requirement for components generated by live bacterial metabolism for optimal fitness [32, 33]. While *E. coli* does not constitute a conventional microbiota, the intimate association of *C. elegans* with bacterial metabolites throughout its lifespan provides a useful and relevant model to examine the impact of bacterial metabolism on host health [34, 35].

**Fig. 1:**
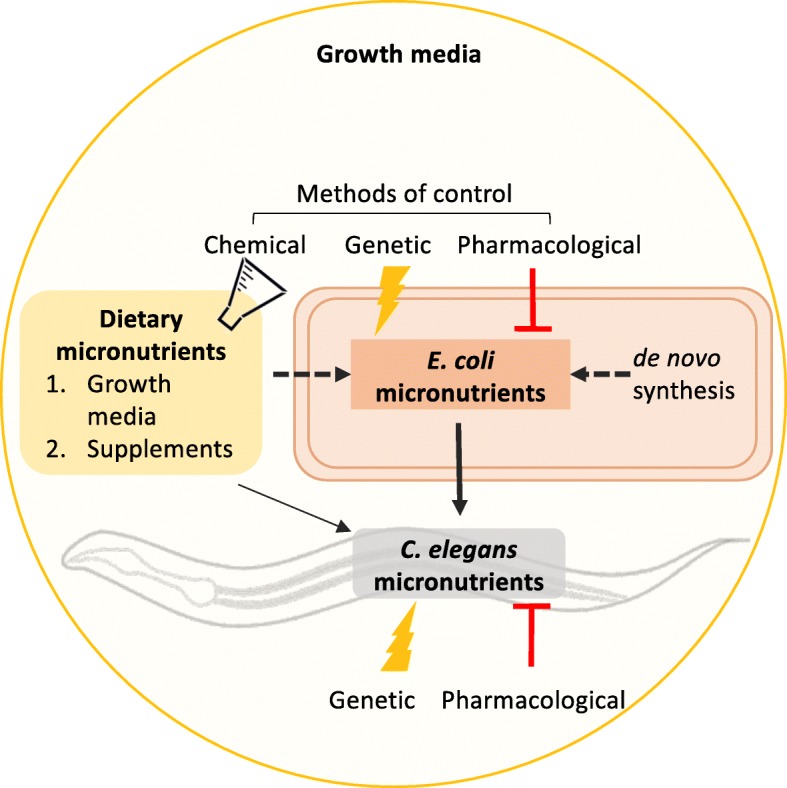
*C*. *elegans*-*E. coli* as a host-microbe model to study the impact of dietary and bacterial micronutrients on host health. *C. elegans* acquires micronutrients primarily through the ingestion of *E. coli* but can also uptake dietary micronutrients directly from the growth media. Like all bacteria, *E. coli* synthesizes some micronutrients de novo and uptakes other micronutrients, or their precursors, from its environment. Little is known about how bacteria interact with dietary micronutrients in the provision of micronutrients to the host. In this model system, the supply of micronutrients to *C. elegans* can be controlled: genetic and pharmacological methods can be used to target specific pathways in both *E. coli* and *C. elegans* and the growth media can be chemically defined

In the experimental system normally used for *C. elegans*, *E. coli* growth is supported by a rich peptone-based growth medium, containing a mixture of peptides, fatty acids, simple carbohydrates, salts, and also trace micronutrients [36]. While *E. coli* is able to synthesize some micronutrients de novo, such as folate, it relies on the uptake of others from the growth medium, including vitamin B12. *E. coli* is unable to take up intact folate, but it can scavenge folate breakdown products from the growth media via a specialized transporter [37]. In our laboratory, we have replaced peptone with a chemically defined medium. Combined with the ability to genetically manipulate both organisms, we have a highly controllable model where we can examine how dietary (from the growth media), bacterial, and supplementary micronutrients interact to impact host health (Fig. 1) [35]. Studies using *C. elegans* have provided novel insights into how bacterial folate synthesis can be both beneficial and detrimental on host health depending on developmental stage and how bacteria act as conduits for folic acid, B12, and iron uptake. This review will examine these studies and discuss the implications of these findings for human health.

## Main text

### Bacterial folate is essential for *C. elegans* development and reproduction

**Fig 2:**
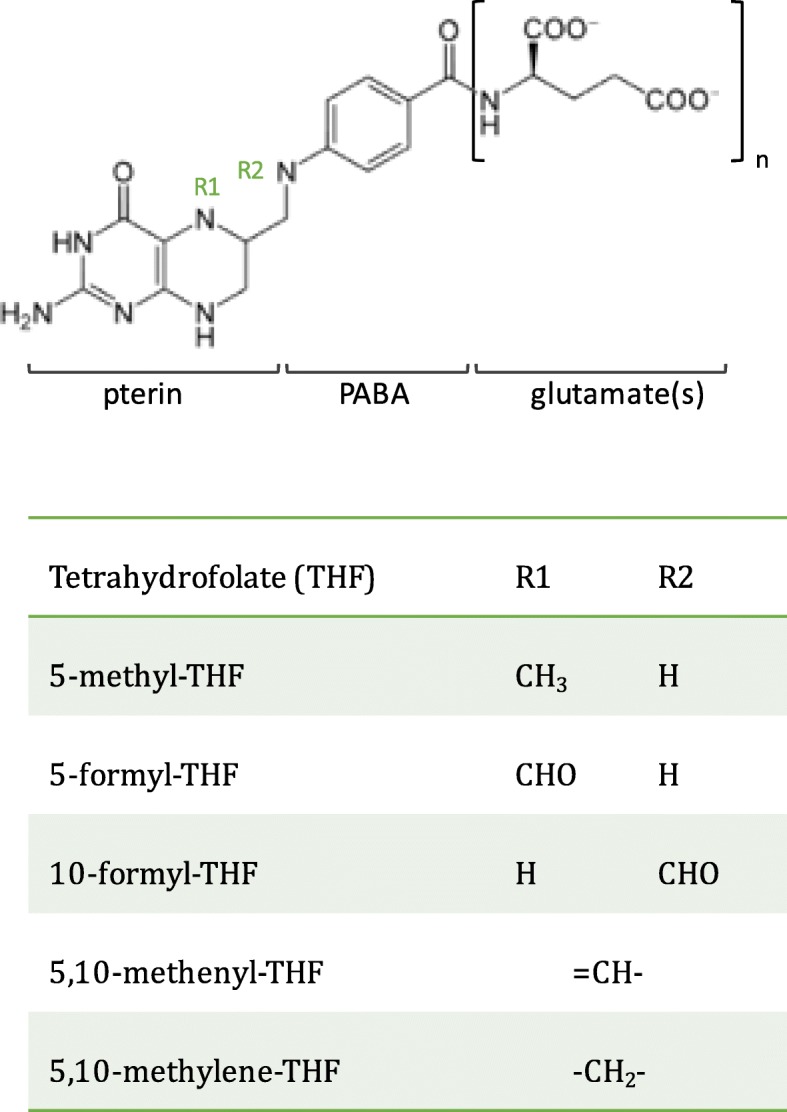
Structure of tetrahydrofolate (THF). THFs are composed of pterin, PABA, and glutamate. The pterin moiety is substituted at N5 (R1) and/or N10 (R2) nitrogen atoms with either a methyl (-CH3), formyl (-CH=O) methylene (=CH2), or methenyl (-CH4) group. THFs also vary in the length of their glutamate “tail,” which varies depending on function

**Fig. 3:**
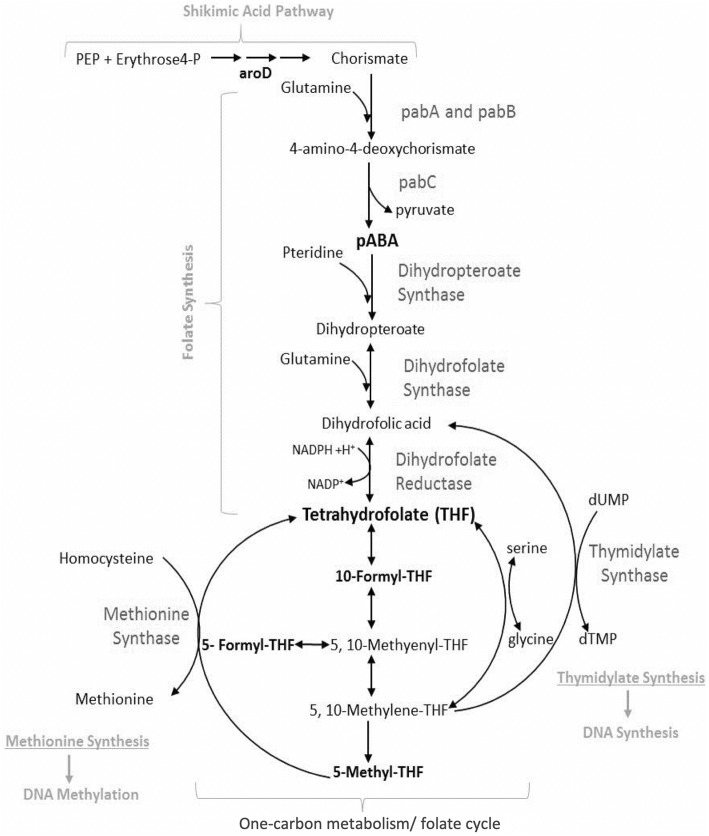
*E. coli* tetrahydrofolate synthesis and the one-carbon metabolism folate cycle. The THF precursors, para-aminobenzoic acid (PABA) (synthesized from chorismate and glutamine), and pteridine (synthesized from GTP) are conjugated to form dihydropteroate, which is glutamylated and reduced to form THF. THF derivatives are generated by the acceptance and transfer of one-carbon units in reactions that facilitate cellular biosynthesis and methylation reactions

Folates are a family of interconvertible water-soluble molecules based on tetrahydrofolate (THF) (Fig. 2) and are used as enzymatic cofactors in a series of reactions known as one-carbon metabolism [38]. Otherwise referred to as the folate cycle, these reactions maintain the pools of several fundamental cellular building blocks, including purines, thymidylate, formylated methionyl-tRNA, methionine, glycine, and serine. Along with an estimated 80% of bacterial species in the human gut microbiome [8, 9], *E. coli* synthesizes THF de novo from the starting materials of para-aminobenzoic acid (PABA) and GTP (Fig. 3). *C. elegans* uptake THFs from the intestine via a reduced folate carrier (RFC), FOLT-1, which shares 40% sequence identity with human RFC [39]. Functional tests with the *C. elegans folt-1* loss-of-function allele, *folt-1*(*ok1560*), reported a massive reduction in fertility caused by defects in spermatogenesis and oogenesis and an associated decrease in the numbers of germline nuclei, in line with the role of one-carbon metabolism in growth and development [40]. *folt-1*(*ok1560*) also decreased both defecation rate and lifespan, however, as the allele was not outcrossed and background mutations could not be ruled out, it was not clear whether these phenotypes were a result of lowered folate status. Chaudhari et al. subsequently examined whether specific bacterial folates or precursors could rescue the germline defect of *folt-1* knockout animals [41]. The addition of the folate precursor, PABA, to the growth medium increased numbers of germ cells and completely rescued fertility in *folt-1* mutants. Supplementation of 10 μM of a purified bacterially derived folate, 5-formyl-THF (folinic acid) to the growth medium rescued the phenotype of *folt-1* mutants fed heat-killed *pabC* mutant *E. coli* (defective in PABA synthesis), therefore demonstrating that the *folt-1* mutant is truly a model of folate deficiency [41]. Together, these studies established *C. elegans* as a model to study folate uptake and metabolism.

Chaudhari et al. used a novel in vitro *C. elegans* germline stem cell culture isolated from a germline tumor mutant strain to examine the impact of specific bacterial folates. By measuring replication of the tumorous germline stem cells and adding specific folates to the culture medium, only 10-formyl-THF and 5,10-methenyl-THF (which rapidly converts to 10-formyl-THF under the experimental pH) were found to be stimulatory folates and at the low concentration of 1 nM. No other THFs tested, including 5-formyl-THF, nor the synthetic supplement folic acid stimulated proliferation, indicating that 10-formyl-THF acts as a specific signal for germline proliferation at a concentration much lower than that required for one-carbon metabolism [41]. Intriguingly, a purified extract of folates from the mouse microbiota was also a potent stimulator of proliferation. The stimulation of cell replication was found to be dependent on the *C. elegans* folate receptor ortholog, FOLR-1, an intriguing finding as the expression of the human folate receptor, FRα is associated with poor prognosis in several cancers [42]. Together, this work raises the possibility that specific bacterially derived folates can act in the host both as micronutrients for one-carbon metabolism and also as signaling molecules for other cellular processes.

### Bacterial folate synthesis accelerates *C. elegans* aging

In contrast to its beneficial role in development, bacterial folate synthesis has been identified as a process which negatively influences *C. elegans* longevity. Initially, this was discovered serendipitously as a result of a spontaneous *E. coli* mutation that substantially increased the lifespan of long-lived *daf-2* mutants [43]. Virk and colleagues isolated the mutation in the *aroD* gene*,* which encodes 3-dehydroquinate dehydratase, an enzyme in the shikimic acid pathway responsible for the synthesis of chorismate. Chorismate is the precursor to several aromatic compounds, including PABA, the precursor of folate synthesis. Lifespan extension on the *aroD E. coli* deletion mutant was reversed only with supplementation of shikimic acid or PABA into the growth media [43]. The sulfonamide drug, sulfamethoxazole (SMX), which acts as a folate synthesis inhibitor by competitively inhibiting PABA production in bacteria [44], was also found to significantly increase *C. elegans* longevity, in a dose-dependent manner, between 1 and 256 μg/ml, without affecting bacterial growth or *C. elegans* development or fecundity [43]. Both PABA and folic acid supplementation reversed longevity on SMX, confirming that this effect was folate-specific. By designing a set of experiments in order to specifically restrict and supplement *C. elegans* folate, it was concluded that animal folate did not impact lifespan. Together, this work identified that bacterial folate, above the level at which it is required to support growth and development, has a detrimental effect on lifespan through an indirect and as yet unidentified effect on bacteria [45].

Two independent screens for *E. coli* mutants that increase *C. elegans* longevity support the hypothesis that bacterial folate synthesis has negative consequences on aging [45, 46]. Of the nine genes isolated in the Virk et al. screen, two were involved in folate synthesis (*pabA* and *pabB*) and three (*pabB*, *aroD*, *aroG*) of the 29 isolated in the Han et al. screen. Notably, neither screen isolated genes involved in folate one-carbon metabolism. Virk et al. noted that *C. elegans* behavioral aversion to the bacterial lawn was increased on *pabA* and *pabB* mutants and on SMX-treated *E. coli*, compared with control conditions, and the authors postulated that excessive bacterial folate synthesis could be associated with a bacterial toxicity. Removal of excessive bacterial folate synthesis, without affecting the level of folate required by *C. elegans* for one-carbon metabolism, increases lifespan [45]. Further study is required to characterize the source of this folate-dependent bacterial toxicity. Han et al. aimed to characterize the host pathways underpinning lifespan extension on their isolated *E. coli* mutants. Lifespan extension on *pabB*, *aroD*, and *aroG* was found to be dependent on the *C. elegans* gene *rict-1*, which encodes a component of the target of rapamycin complex 2 (TORC2), known to interact with the insulin signaling pathway and integrate nutritional signals to extend lifespan [47]. Lifespan was not dependent on *eat-2* or *daf-16*, thus ruling out dietary restriction as a mechanism for lifespan increase [46]. No further investigation was carried out into the interaction of bacterial folates with TORC2, and the mechanism underpinning how folate acts as a nutritional signal to *C. elegans* is unclear.

An associated study reported that the biguanide drug, metformin, could modulate *C. elegans* lifespan by disrupting bacterial folate synthesis [34]. Metformin, a mitochondria complex-1 inhibitor, had previously been identified to extend *C. elegans* lifespan, however, it had been assumed it was directly acting on host targets [48]. Cabreiro and colleagues demonstrated that a high concentration of metformin (50 mM) increased lifespan on certain strains of live *E. coli* but conversely shortened lifespan when *C. elegans* were raised on UV-killed *E. coli* or on axenic medium. Metformin also decreased the lifespan of *C. elegans* fed bacteria resistant to metformin. Therefore, lifespan extension was dependent on bacterial metabolism of metformin, but in the absence of this interaction, metformin at this concentration was directly toxic to the worm. Analysis of bacterial metabolites following metformin treatment indicated that lifespan extension was associated with decreased levels of nearly all folates but increased levels of 5-methyl-THF. The authors hypothesized that this could occur following inhibition of the enzyme, methionine synthase, which uses 5-methyl-THF as a cofactor to convert homocysteine to methionine; however, neither methionine levels nor transcript levels of methionine synthase were shown. Levels of the methyl donor, S-adenosyl-methionine (SAM), which is synthesized from methionine, were significantly lower in *C. elegans* following metformin treatment but was higher in *E. coli.* Metformin could not increase the lifespan of the long-lived *C. elegans sams-1*(*ok3033*) mutant and in fact significantly decreased lifespan in this background. As metformin lifespan extension had previously been reported to be dependent on the AMP-dependent protein kinase (AMPK) [48], whether *sams-1* RNAi could extend the lifespan of the short-lived *aak-2* (*ok524*) mutant was tested. No effect was reported, suggesting that metformin and methionine restriction may be acting via AMPK to extend lifespan. This study therefore demonstrated that the longevity extension observed with metformin was at least in part mediated by bacteria via disruption to bacterial folate metabolism. The impact of both metformin and sulfamethoxazole on longevity is dependent on the alteration of bacterial folate metabolism.

### A bacterial route of folic acid supplementation

A significant finding in the above body of work was that folic acid, a synthetic folate supplement, decreased the lifespan of long-lived *C. elegans* on folate-inhibited *E. coli* [43]. This was intriguing as the data had shown that supplementing host folate did not have a negative impact on longevity. We know from early microbiological studies that *E. coli* cannot uptake intact folic acid, but could folic acid supplementation somehow be boosting bacterial folate synthesis? In order to answer this question, the group characterized a genetic means to differentiate between host and bacterial folate. In humans, an intestinally expressed glutamate carboxypeptidase is required for the cleavage of polyglutamated THFs into monoglutamated THFs, the preferred substrate for RFCs. Most dietary and bacterial folates are polyglutamates with a chain of up to eight residues (Fig. 2); polyglutamation is thought to favor cellular retention and increase affinity for folate-dependent enzymes, whereas folate transporters favor monoglutamation [49–51]. The *C. elegans* ortholog, *gcp-2.1*, was characterized and found to be necessary for the generation of monoglutamated THFs [45]*.* Worms homozygous for the *gcp-2.1* (*ok1004*) loss-of-function allele showed no phenotypic signs of folate deficiency under standard culture conditions; however, when raised on *pabA E. coli* (defective in PABA synthesis) or *E. coli* treated with SMX, *gcp-2.1* (*ok1004*) worms were severely developmentally delayed and were sterile. Together, this showed that under standard culture conditions, *E. coli* synthesizes sufficient monoglutamated THF to support one-carbon metabolism, but when this supply falls below a certain threshold GCP-2.1 activity is required.

As the *gcp-2.1* mutant is phenotypically responsive to bacterial folate levels, it was harnessed as a means to determine whether folic acid supplementation altered bacterial folate synthesis. A synthetic defined folate-free growth media was used to eliminate the impact of exogenous folates or precursors on either organism. Using this controlled system, supplementation of folic acid into the defined media was found to rescue the *gcp-2.1* phenotype on SMX-treated *E. coli* at concentrations ten-fold higher than that of folinic acid, indicating two different mechanisms of uptake in *C. elegans* for naturally occurring THFs and folic acid [45]. The assay was repeated on *pabA* mutant *E. coli*, which has the same effect on *gcp-2.1* development as SMX [52]. Surprisingly, it was found that folic acid rescue was dependent on the *E. coli* transporter, *abgT*, specialized for the uptake of the folate breakdown product PABA-glutamate (PABA-glu). In contrast, rescue with folinic acid was independent of *abgT* expression. LC-MS/MS analysis of several folic acid preparations, including a commercial folic acid supplement, revealed the presence of the breakdown products PABA-glu and PABA at physiological concentrations [5].

This bacterial route of folic acid supplementation was also shown to be responsible for shortening the lifespan of worms on folate-deficient *pabA* mutant bacteria. Ten micromolar folic acid reversed the increased lifespan of these worms, but in a manner dependent on the bacterial *abgT* gene, demonstrating that bacterial uptake of the folic acid breakdown product, PABA-glu was required [52]. Folinic acid, on the other hand, did not decrease lifespan at this concentration. Measuring bacterial folate levels by LC-MS/MS following folic acid supplementation (10 μM) revealed an increase in folate levels in *pabA* but not *abgTpabA E. coli* [52]. Folic acid therefore indirectly supplements *C. elegans* by boosting bacterial synthesis of tetrahydrofolates, which are more readily absorbed in the nematode intestine than the synthetic supplement itself.

Together, this work presents an alternative and unacknowledged route by which bacteria may increase the bioavailability of folic acid to their host by the indirect uptake of breakdown products and synthesis of THFs by bacteria, which are more readily absorbed in the intestine. While this pathway has a beneficial impact on the host during development, it may accelerate aging of the host.

### *E. coli* acts as a conduit for vitamin B12 uptake

Vitamin B12 has also been widely studied using the *C. elegans-E. coli* model system. In animals, B12 is required for the interconversion of homocysteine to methionine and the metabolism of carbon sources to succinyl-CoA to feed into the TCA cycle. B12 is synthesized exclusively by archaea and a small proportion of bacteria in a complex biosynthetic pathway [9]. *E. coli* is unable to synthesize vitamin B12 and instead scavenges exogenous B12 via a siderophore complex dependent on *tonB* [53]*.* This pathway has been shown to be vital for the uptake of B12 by *C. elegans*; a *tonB* mutant induces the expression of *acdh-1p::GFP* in *C*. *elegans,* (GFP under the control of the acyl-CoA dehydrogenase promoter) a dietary sensor of B12 deficiency [54], indicating that *C. elegans* is unable to uptake sufficient B12 directly from the growth media. *E. coli* therefore acts as a conduit for the provision of bioavailable B12 to *C. elegans.* The importance of this bacterial route is questioned, as expression of *acdh-1p::GFP* on *E. coli* was found to be suppressed with supplementation of B12 on both live and UV-killed bacteria [54]. The reason for this discrepancy is perhaps due to the increased bioavailability of B12 in a preparation of a B12 supplement compared with B12 present in trace amounts in the growth media.

*C. elegans* continues to be a useful model to study B12 deficiency [55]. Worms exhibit infertility, developmental growth defects, memory impairment, and a reduced lifespan when maintained on *E. coli* on B12-deficient growth media [56, 57]. Several studies have provided insights into novel roles of vitamin B12 on host metabolism and immunity, which have conserved role in humans [58–60]. Furthermore, *C. elegans* can be used as a phenotypic readout of bacterial B12 status: expression of *acdh-1p::GFP* on the B12-synthesizing bacterium, *Comamonas aquatica*, is significantly lower when compared with worms on the standard *E. coli* diet [54]. This provides a rapid and low-cost screen for the bacterial production of bioavailable B12 which may be useful for the development of novel probiotic micronutrient solutions.

### Bacterial siderophore associates with host to assist iron uptake

Mammalian studies investigating the interaction of gut bacteria in the uptake of iron have mostly focused on the tug-of-war between pathogenic microbes and host; enterobacteria secrete enterobactin (Ent), a siderophore with a high affinity for iron which scavenges iron from host mitochondria [25], and the host fights back against this “iron piracy” by secreting siderocalin which binds and sequesters Ent [61]. How the host differentiates between commensal and pathogenic bacteria, both of which secrete enterobactins, remained unexplored. A recent study using the *C. elegans*-*E. coli* host-microbe model demonstrated that commensal bacteria, such as non-pathogenic *E. coli*, which also synthesize Ent, are actually beneficial to host iron homeostasis [62]. Qi et al. carried out a screen for *E. coli* genes required for *C. elegans* development and isolated five *E. coli* mutants defective in the synthesis of Ent. Supplementation with Ent was able to rescue host development on a restricted diet of Ent deficient *E. coli.* By using affinity purification and measuring radiolabelled ^55^Fe in host mitochondria, the group demonstrated that Ent associated with the α-subunit of the host ATP synthase and that both of these components were required for the increase in levels of iron in the host mitochondria.

The group proposed a model whereby enterobactin released from commensal bacteria scavenges exogenous iron which facilitates uptake in the host mitochondria. It was implied that this occurs within the *C. elegans* intestine, with Ent-Fe^3+^ directly entering the host mitochondria. Addition of Ent to heat-killed *E. coli* also increased host iron levels, eliminating the possibility that the benefit of Ent on host iron is indirect via initial uptake of iron by *E. coli. E. coli* uptake of Ent-Fe is TonB-dependent; it would be interesting to test whether host iron levels were increased to the same extent in a *tonB* mutant following Ent supplementation, in order to cement the model. Encouragingly, the group demonstrated the same role of Ent in a mammalian cell culture [62]. The discovery of this mechanism in *C. elegans* is a testament to the flexibility and simplicity of the host-microbe model system.

## Conclusions

### Implications for human health

The advancement in metagenomic sequencing technologies has enabled the description of the micronutrient biosynthetic capacity of the human gut microbiota, but there is a substantial lack of mechanistic studies examining whether this contributes significantly to host micronutrient status and whether dietary and supplemental micronutrients interact with bacterial metabolism. While a simplified invertebrate model system cannot be used to make quantitative conclusions on human micronutrient demand and intake, it can be useful in understanding the qualitative mechanisms in which bacteria play a role in the provision of micronutrients and the effects that these have on health [63].

#### Folate

Bacterial folate acts as an essential micronutrient for one-carbon metabolism during development and as a germline signal during development in *C. elegans* [41]. In contrast, bacterial folate synthesis, above the threshold required to support one-carbon metabolism, has a negative impact on host longevity [45]. Together, this work indicates that interventions that disrupt bacterial folate synthesis may have implications on “stimulatory folate signaling” to the germ line, which can result in cancerous proliferation [41] and aging [45]. In humans, bacterial folate synthesis in the microbiota changes with developmental stage, with an upregulation of folate synthesizing genes in babies compared with adults and an upregulation of folate-salvaging genes in adults compared with babies [64]. Aging is often associated with an increase in pro-inflammatory Proteobacteria, a predicted 71% of which synthesize folate de novo [8]*.* Whether aging is associated with an increase in colonic folate remains to be determined. Interestingly, a sulfonamide drug, sulfadiazine, has previously been shown to extend rodent lifespan [65]. Although this study did not mention the impact on the mouse microbiota, PABA supplementation reversed lifespan. As PABA cannot be metabolized by mammals, this points to a role of inhibiting bacterial folate synthesis in lifespan extension. Whether bacterial folate synthesis in the human gut microbiome may represent a novel target to slow down the aging process warrants further study [66].

#### Folic acid

*E. coli* uptake of the folic acid breakdown product PABA-glu was found to be the dominant route by which *C. elegans* takes up folic acid [52]. In humans, folic acid is predicted to be absorbed in the small intestine by the proton-coupled folate transporter (PCFT), however, the folate breakdown product PABA has been identified as a human excretory product following folic acid supplementation, indicating that it does reach the colon. Infusion of [^3^H] PABA into the colon of rats [67] and piglets [68] could be traced to THFs in the host, demonstrating that bacterial uptake of PABA generates THFs that are absorbed and used in one-carbon metabolism. The lack of regulation of micronutrient supplements but fierce competition on the market has led to a compromise on quality, and folic acid supplements are commonly found to fail tests of stability and dissolution [69–72]. Combined with the instability of folic acid at low pH [73], it is likely that folic acid breakdown products will be available to the colonic microbiota following long-term supplementation. There have been no studies examining the impact of long-term folic acid supplementation on the microbiota. It is of note that bacteria unable to synthesize folate de novo but able to scavenge folate breakdown products via AbgT are commonly pathogenic and pro-inflammatory Proteobacteria associated with an elderly microbiome [74]. The naturally occurring folate, folinic acid, which was not found to impact bacterial metabolism to the same extent as folic acid [52] may be a better alternative to folic acid as a folate supplement. Further studies are required to determine how different folate supplements influence microbial metabolism, composition, and long-term host health in mammalian models with more complex microbiotas.

#### B12

In humans, the absorption of B12 requires hydrolysis of dietary protein-bound B12 by stomach acids and association with a host-derived intrinsic factor (IF) in the small intestine [75]. There is a lack of evidence that B12 from the pool of bacterially derived B12 in the human colon is absorbed. Instead, B12 in the colon is predicted to act as a keystone metabolite which regulates the composition of the microbiota [76]. Patients with small intestinal bacterial overgrowth often suffer with B12 deficiency, likely due to the association of bacteria with IF-B12 complexes [77]. IF is not coded for in the *C. elegans* genome, but uptake is at least partially dependent on bacterial *tonB*, part of the complex that transports B12 into *E. coli*. From this work, it is tempting to speculate that commensal bacteria may assist in the bioavailability of B12 to the host under certain conditions, perhaps by the association with a bacterial *tonB-*dependent intrinsic factor which facilitates host uptake.

#### Iron

Bacteria are thought to compete with the host for iron uptake. The work by Qi et al. demonstrates that commensal bacteria can facilitate host uptake of iron via the secretion of enterobactin. Whether commensal Ent works in this way in an in vivo mammalian model is yet to be established, however, this work provides the intriguing possibility that Ent could be harnessed as a novel molecule to increase the efficiency of iron supplementation in the treatment of iron deficiency.

### Concluding remarks

**Fig. 4:**
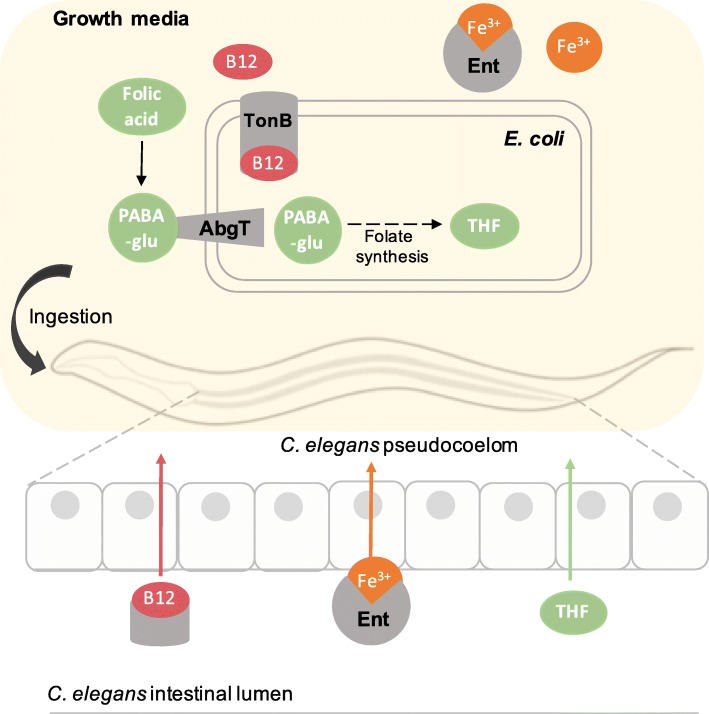
*E. coli* assists *C. elegans* in the uptake of micronutrients. Supplementation of folic acid to *C. elegans* is dependent on bacterial uptake of the breakdown product, PABA-glu, which is used as a precursor to generate tetrahydrofolate (THF). THFs were found to be more effectively absorbed than intact folic acid by *C. elegans.* B12 supplementation is dependent on the bacterial TonB-complex, indicating that a bacterial factor increases the bioavailability of B12. Iron supplementation is boosted by the bacterial siderophore, enterobactin, which associates with host mitochondria when bound to Fe^3+^ ions

Several bacterial mechanisms that play a role in the effective supplementation of micronutrients, either by the secretion of siderophores (iron and B12), or the uptake and metabolism into a more readily absorbable derivative (folic acid), have been revealed using *C. elegans-E. coli* as a host-microbe system (Fig. 4). These mechanisms act to make the micronutrient more bioavailable to the host but, in the case of folic acid, may have negative implications for long-term health under certain conditions. Micronutrients are emerging as core regulators of microbiome stability, and further study into the potential disruption caused by long-term micronutrient supplementation on the microbiota are called for. Together, these studies warrant further research using models with more complex microbiotas on the role of bacteria in the uptake of micronutrients and the implications on host health. Understanding these interactions will help us to design more effective interventions to boost micronutrient uptake in the GI tract.

## Data Availability

Not applicable

## Abbreviations

THF: Tetrahydrofolate
PABA: Para-aminobenzoic acid
PABA-glu: PABA-glutamate/para-aminobenzoylglutamate

## References

[1] Biesalski HK (2016). Nutrition meets the microbiome: micronutrients and the microbiota. Ann N Y Acad Sci..

[2] Kim TH, Yang J, Darling PB, O'Connor DL (2004). A large pool of available folate exists in the large intestine of human infants and piglets. J Nutr..

[3] O'Keefe SJ, Ou J, Aufreiter S, O'Connor D, Sharma S, Sepulveda J (2009). Products of the colonic microbiota mediate the effects of diet on colon cancer risk. J Nutr..

[4] Said HM, Mohammed ZM (2006). Intestinal absorption of water-soluble vitamins: an update. Curr Opin Gastroenterol..

[5] Aufreiter S, Gregory JF, Pfeiffer CM, Fazili Z, Kim YI, Marcon N (2009). Folate is absorbed across the colon of adults: evidence from cecal infusion of (13)C-labeled [6S]-5-formyltetrahydrofolic acid. Am J Clin Nutr..

[6] Lakoff A, Fazili Z, Aufreiter S, Pfeiffer CM, Connolly B, Gregory JF (2014). Folate is absorbed across the human colon: evidence by using enteric-coated caplets containing 13C-labeled [6S]-5-formyltetrahydrofolate. The American Journal of Clinical Nutrition..

[7] Chan YM, Aufreiter S, O'Keefe SJ, O'Connor DL (2019). Switching to a fibre-rich and low-fat diet increases colonic folate contents among African Americans. Appl Physiol Nutr Metab..

[8] Magnusdottir S, Ravcheev D, de Crecy-Lagard V, Thiele I (2015). Systematic genome assessment of B-vitamin biosynthesis suggests co-operation among gut microbes. Front Genet..

[9] Rodionov DA, Arzamasov AA, Khoroshkin MS, Iablokov SN, Leyn SA, Peterson SN (2019). Micronutrient requirements and sharing capabilities of the human gut microbiome. Front Microbiol..

[10] Hibbard BM, Hibbard ED, Jeffcoate TN (1965). Folic acid and reproduction. Acta Obstet Gynecol Scand..

[11] Hibbard BM (1964). The role of folic acid in pregnancy; with particular reference to anaemia, abruption and abortion. J Obstet Gynaecol Br Commonw..

[12] Zsigrai S, Kalmar A, Valcz G, Szigeti KA, Bartak BK, Nagy ZB (2019). Physiological and pathophysiological significance of vitamin B9. Summary on the occasion of the 30-year introduction of folic acid as a dietary supplement. Orv Hetil..

[13] Honein MA, Paulozzi LJ, Mathews TJ, Erickson JD, Wong LY (2001). Impact of folic acid fortification of the US food supply on the occurrence of neural tube defects. JAMA..

[14] Sayed AR, Bourne D, Pattinson R, Nixon J, Henderson B (2008). Decline in the prevalence of neural tube defects following folic acid fortification and its cost-benefit in South Africa. Birth Defects Res A Clin Mol Teratol..

[15] Bower C, D'Antoine H, Stanley FJ (2009). Neural tube defects in Australia: trends in encephaloceles and other neural tube defects before and after promotion of folic acid supplementation and voluntary food fortification. Birth Defects Res A Clin Mol Teratol..

[16] Milne DB, Canfield WK, Mahalko JR, Sandstead HH (1984). Effect of oral folic acid supplements on zinc, copper, and iron absorption and excretion. Am J Clin Nutr..

[17] Selhub J, Morris MS, Jacques PF, Rosenberg IH (2009). Folate-vitamin B-12 interaction in relation to cognitive impairment, anemia, and biochemical indicators of vitamin B-12 deficiency. Am J Clin Nutr..

[18] Cole BF, Baron JA, Sandler RS, Haile RW, Ahnen DJ, Bresalier RS (2007). Folic acid for the prevention of colorectal adenomas: a randomized clinical trial. JAMA..

[19] Marean A, Graf A, Zhang Y, Niswander L (2011). Folic acid supplementation can adversely affect murine neural tube closure and embryonic survival. Hum Mol Genet..

[20] Pickell L, Brown K, Li D, Wang XL, Deng L, Wu Q (2011). High intake of folic acid disrupts embryonic development in mice. Birth Defects Res A Clin Mol Teratol.

[21] Carroll C, Cooper K, Papaioannou D, Hind D, Tappenden P, Pilgrim H (2010). Meta-analysis: folic acid in the chemoprevention of colorectal adenomas and colorectal cancer. Aliment Pharmacol Ther..

[22] Fife J, Raniga S, Hider PN, Frizelle FA (2011). Folic acid supplementation and colorectal cancer risk: a meta-analysis. Colorectal Dis..

[23] Qin T, Du M, Du H, Shu Y, Wang M, Zhu L (2015). Folic acid supplements and colorectal cancer risk: meta-analysis of randomized controlled trials. Sci Rep..

[24] Kok DE, Steegenga WT, Smid EJ, Zoetendal EG, Ulrich CM, Kampman E. Bacterial folate biosynthesis and colorectal cancer risk: more than just a gut feeling. Crit Rev Food Sci Nutr. 2018:1–13. 10.1080/10408398.2018.1522499 30501511

[25] Yilmaz Bahtiyar, Li Hai (2018). Gut Microbiota and Iron: The Crucial Actors in Health and Disease. Pharmaceuticals.

[26] Seril DN, Liao J, Ho KL, Warsi A, Yang CS, Yang GY (2002). Dietary iron supplementation enhances DSS-induced colitis and associated colorectal carcinoma development in mice. Dig Dis Sci..

[27] Erichsen K, Milde AM, Arslan G, Helgeland L, Gudbrandsen OA, Ulvik RJ (2005). Low-dose oral ferrous fumarate aggravated intestinal inflammation in rats with DSS-induced colitis. Inflamm Bowel Dis..

[28] Lee T, Clavel T, Smirnov K, Schmidt A, Lagkouvardos I, Walker A (2017). Oral versus intravenous iron replacement therapy distinctly alters the gut microbiota and metabolome in patients with IBD. Gut..

[29] Jaeggi T, Kortman GA, Moretti D, Chassard C, Holding P, Dostal A (2015). Iron fortification adversely affects the gut microbiome, increases pathogen abundance and induces intestinal inflammation in Kenyan infants. Gut..

[30] Zecic A, Dhondt I, Braeckman BP (2019). The nutritional requirements of Caenorhabditis elegans. Genes Nutr..

[31] Garigan D, Hsu AL, Fraser AG, Kamath RS, Ahringer J, Kenyon C (2002). Genetic analysis of tissue aging in Caenorhabditis elegans: a role for heat-shock factor and bacterial proliferation. Genetics..

[32] Gems D, Riddle DL (2000). Defining wild-type life span in Caenorhabditis elegans. J Gerontol A Biol Sci Med Sci..

[33] Lenaerts I, Walker GA, Van Hoorebeke L, Gems D, Vanfleteren JR (2008). Dietary restriction of Caenorhabditis elegans by axenic culture reflects nutritional requirement for constituents provided by metabolically active microbes. J Gerontol A Biol Sci Med Sci..

[34] Cabreiro F, Au C, Leung KY, Vergara-Irigaray N, Cocheme HM, Noori T (2013). Metformin retards aging in C. elegans by altering microbial folate and methionine metabolism. Cell..

[35] Yilmaz LS, Walhout AJ (2014). Worms, bacteria, and micronutrients: an elegant model of our diet. Trends Genet..

[36] Brenner S (1974). The genetics of Caenorhabditis elegans. Genetics..

[37] Carter EL, Jager L, Gardner L, Hall CC, Willis S, Green JM (2007). Escherichia coli abg genes enable uptake and cleavage of the folate catabolite p-aminobenzoyl-glutamate. J Bacteriol..

[38] Fox JT, Stover PJ (2008). Folate-mediated one-carbon metabolism. Vitam Horm..

[39] Balamurugan K, Ashokkumar B, Moussaif M, Sze JY, Said HM (2007). Cloning and functional characterization of a folate transporter from the nematode Caenorhabditis elegans. Am J Physiol Cell Physiol..

[40] Austin MU, Liau WS, Balamurugan K, Ashokkumar B, Said HM, LaMunyon CW (2010). Knockout of the folate transporter folt-1 causes germline and somatic defects in C. elegans. BMC Dev Biol..

[41] Chaudhari SN, Mukherjee M, Vagasi AS, Bi G, Rahman MM, Nguyen CQ (2016). Bacterial folates provide an exogenous signal for C. elegans germline stem cell proliferation. Dev Cell..

[42] Cheung A, Bax HJ, Josephs DH, Ilieva KM, Pellizzari G, Opzoomer J (2016). Targeting folate receptor alpha for cancer treatment. Oncotarget..

[43] Virk B, Correia G, Dixon DP, Feyst I, Jia J, Oberleitner N (2012). Excessive folate synthesis limits lifespan in the C. elegans: E. coli aging model. BMC Biol.

[44] Seydel JK (1968). Sulfonamides, structure-activity relationship, and mode of action. Structural problems of the antibacterial action of 4-aminobenzoic acid (PABA) antagonists. J Pharm Sci..

[45] Virk B, Jia J, Maynard CA, Raimundo A, Lefebvre J, Richards SA (2016). Folate acts in E. coli to accelerate C. elegans aging independently of bacterial biosynthesis. Cell Rep..

[46] Han B, Sivaramakrishnan P, Lin CJ, Neve IAA, He J, Tay LWR (2017). Microbial genetic composition tunes host longevity. Cell..

[47] Soukas AA, Kane EA, Carr CE, Melo JA, Ruvkun G (2009). Rictor/TORC2 regulates fat metabolism, feeding, growth, and life span in Caenorhabditis elegans. Genes Dev..

[48] Onken B, Driscoll M (2010). Metformin induces a dietary restriction-like state and the oxidative stress response to extend C. elegans healthspan via AMPK, LKB1, and SKN-1. PLoS One.

[49] Egan MG, Sirlin S, Rumberger BG, Garrow TA, Shane B, Sirotnak FM (1995). Rapid decline in folylpolyglutamate synthetase activity and gene expression during maturation of HL-60 cells. Nature of the effect, impact on folate compound polyglutamate pools, and evidence for programmed down-regulation during maturation. J Biol Chem..

[50] Schirch V, Strong WB (1989). Interaction of folylpolyglutamates with enzymes in one-carbon metabolism. Arch Biochem Biophys..

[51] Turner FB (1999). Andreassi 2nd JL, Ferguson J, Titus S, Tse A, Taylor SM, et al. Tissue-specific expression of functional isoforms of mouse folypoly-gamma-glutamae synthetase: a basis for targeting folate antimetabolites. Cancer Res..

[52] Maynard C, Cummins I, Green J, Weinkove D (2018). A bacterial route for folic acid supplementation. BMC Biol..

[53] Kadner RJ (1990). Vitamin B12 transport in Escherichia coli: energy coupling between membranes. Molecular microbiology..

[54] Watson E, MacNeil LT, Ritter AD, Yilmaz LS, Rosebrock AP, Caudy AA (2014). Interspecies systems biology uncovers metabolites affecting C. elegans gene expression and life history traits. Cell..

[55] Bito T, Watanabe F (2016). Biochemistry, function, and deficiency of vitamin B12 in Caenorhabditis elegans. Exp Biol Med (Maywood)..

[56] Bito T, Matsunaga Y, Yabuta Y, Kawano T, Watanabe F (2013). Vitamin B12 deficiency in Caenorhabditis elegans results in loss of fertility, extended life cycle, and reduced lifespan. FEBS Open Bio..

[57] Bito T, Misaki T, Yabuta Y, Ishikawa T, Kawano T, Watanabe F (2017). Vitamin B12 deficiency results in severe oxidative stress, leading to memory retention impairment in Caenorhabditis elegans. Redox Biol..

[58] Watson E, Olin-Sandoval V, Hoy MJ, Li CH, Louisse T, Yao V, et al. Metabolic network rewiring of propionate flux compensates vitamin B12 deficiency in C. elegans. Elife. 2016:5. 10.7554/eLife.17670 PMC4951191 27383050

[59] Na H, Ponomarova O, Giese GE, Walhout AJM (2018). C. elegans MRP-5 exports vitamin B12 from mother to offspring to support embryonic development. Cell Rep..

[60] Revtovich AV, Lee R, Kirienko NV (2019). Interplay between mitochondria and diet mediates pathogen and stress resistance in Caenorhabditis elegans. PLoS Genet..

[61] Golonka R, Yeoh BS, Vijay-Kumar M (2019). The iron tug-of-war between bacterial siderophores and innate immunity. J Innate Immun..

[62] Qi B, Han M (2018). Microbial siderophore enterobactin promotes mitochondrial iron uptake and development of the host via interaction with ATP synthase. Cell..

[63] Gottschling DC, Doring F (2019). Is C. elegans a suitable model for nutritional science?. Genes Nutr.

[64] Yatsunenko T, Rey FE, Manary MJ, Trehan I, Dominguez-Bello MG, Contreras M (2012). Human gut microbiome viewed across age and geography. Nature..

[65] Hackmann C (1958). Observations on influenceability of age phenomena in experimental animals by peroral administration of combinations of 2-(p-aminobenzolsulfonamide)-pyrimidin. Munch Med Wochenschr..

[66] Maynard C, Weinkove D (2018). The gut microbiota and ageing. Subcell Biochem..

[67] Rong N, Selhub J, Goldin BR, Rosenberg IH (1991). Bacterially synthesized folate in rat large intestine is incorporated into host tissue folyl polyglutamates. J Nutr..

[68] Asrar FM, O'Connor DL (2005). Bacterially synthesized folate and supplemental folic acid are absorbed across the large intestine of piglets. J Nutr Biochem..

[69] Sculthorpe NF, Davies B, Ashton T, Allison S, McGuire DN, Malhi JS (2001). Commercially available folic acid supplements and their compliance with the British Pharmacopoeia test for dissolution. J Public Health Med..

[70] Hoag SW, Ramachandruni H, Shangraw RF (1997). Failure of prescription prenatal vitamin products to meet USP standards for folic acid dissolution. J Am Pharm Assoc (Wash).

[71] Andrews KW, Roseland JM, Gusev PA, Palachuvattil J, Dang PT, Savarala S (2017). Analytical ingredient content and variability of adult multivitamin/mineral products: national estimates for the Dietary Supplement Ingredient Database. Am J Clin Nutr.

[72] Dwyer Johanna, Coates Paul, Smith Michael (2018). Dietary Supplements: Regulatory Challenges and Research Resources. Nutrients.

[73] De Brouwer V, Zhang GF, Storozhenko S, Straeten DV, Lambert WE (2007). pH stability of individual folates during critical sample preparation steps in prevision of the analysis of plant folates. Phytochem Anal..

[74] Claesson MJ, Cusack S, O'Sullivan O, Greene-Diniz R, de Weerd H, Flannery E (2011). Composition, variability, and temporal stability of the intestinal microbiota of the elderly. Proc Natl Acad Sci U S A..

[75] Grasbeck R (1969). Intrinsic factor and the other vitamin B12 transport proteins. Prog Hematol..

[76] Degnan PH, Taga ME, Goodman AL (2014). Vitamin B12 as a modulator of gut microbial ecology. Cell Metab..

[77] Schjonsby H (1989). Vitamin B12 absorption and malabsorption. Gut..

